# Preventing Type 2 Diabetes after Gestational Diabetes: A Systematic Review Mapping Physical Activity Components using the Socio-Ecological Model

**DOI:** 10.1007/s10995-024-03948-w

**Published:** 2024-06-03

**Authors:** Elysa Ioannou, Helen Humphreys, Catherine Homer, Alison Purvis

**Affiliations:** 1 Sport and Physical Activity Research Centre, Sheffield Hallam University, Sheffield, UK; 2 Centre for Behavioural Science and Applied Psychology (CeBSAP), Sheffield Hallam University, Sheffield, UK

**Keywords:** Exercise, Gestational Diabetes Mellitus, Physical Activity, Socio-Ecological Model, Type 2 Diabetes Mellitus

## Abstract

**Objectives:**

Gestational diabetes commonly occurs during pregnancy and increases lifetime risk of type 2 diabetes following pregnancy. Engaging in physical activity postnatally can reduce this subsequent risk. Interventions aiming to increase physical activity after gestational diabetes may not address the wide range of post-pregnancy barriers. A socio-ecological approach highlights the need to include multi-level factors such as social, community and organisational factors. The aim of the review was to map intervention components to prevent type 2 diabetes after gestational diabetes using the socio-ecological model as a framework and investigate how physical activity changes align with different intervention components utilised.

**Methods:**

Eligible studies included any study type within 5 years of a gestational diabetes diagnosis and targeted physical activity. A systematic search of MEDLINE, Cochrane Library, Web of Science, CINAHL Complete, and Scopus was conducted in October 2022. Results were categorised based on whether findings demonstrated no increases, non-statistically significant increases or statistically significant increases in physical activity.

**Results:**

Forty-eight studies were included (37 different interventions). Thirty-eight studies were assessed as “adequate” quality, only two studies were “good” quality, and the remaining were limited quality. Mixed physical activity outcomes were observed across components used at the intrapersonal level, with components across other levels of the socio-ecological model showing more increases in physical activity. Intervention components within the social and organisational levels, for example childcare provision, providing group-based sessions and offering remote delivery, were more often present in interventions with physical activity increases.

**Conclusions for Practice:**

Future interventions targeting physical activity after gestational diabetes should aim to include social and organisational-level components in their intervention design.

This systematic review was registered in PROSPERO (ID: CRD42021272044).

## Significance

**What is Already Known on this Subject?:**

Physical activity can reduce risk of type 2 diabetes after gestational diabetes. However, interventions in this population are not sustainable, have low attendance and high dropout rates.

**What this Study Adds?:**

This review has highlighted intervention components across levels of the socio-ecological model which could have important implications in uptake and maintenance of physical activity after gestational diabetes. Findings should be considered in intervention planning and design, ensuring a combination of multi-level approaches are purposefully included. Examples include providing childcare, facilitating social support through group-based sessions and offering increased flexibility through remote delivery.

## Introduction

Gestational Diabetes Mellitus (GDM) is a common complication in pregnancy, resulting in short- and long-term complications in both women and their infants (Metzger, 2010). One potential long-term complication is the development of Type 2 Diabetes Mellitus (T2DM), where subsequent risk is ten-fold that of women with a normoglycemic pregnancy (Vounzoulaki et al., 2020). Preventing T2DM after GDM is currently recognised as one of the top-ten research priorities, according to literature, Health Care Professionals (HCP) and women who have had GDM (Ayman et al., 2021). The National Institute for Health and Care Excellence (NICE) recommends educating about lifestyle change after a GDM pregnancy to reduce T2DM risk (NICE, 2020).

Lifestyle changes, including physical activity (PA) and dietary changes, have been shown to reduce onset of T2DM by over 50% (Knowler et al., 2002; Pan et al., 1997; Tuomilehto et al., 2001). When these are adopted by women with previous GDM, T2DM development can also be effectively prevented (Bentley-Lewis et al., 2008; Chasan-Taber, 2015). PA alone may independently reduce risk of future T2DM, however is not effectively encouraged after GDM (Bao et al., 2014; Jones et al., 2017a, 2017b). Despite interventions improving dietary behaviour and resulting in weight-loss, challenges in PA uptake remain (Jones et al., 2017a, 2017b). Furthermore, the UK National Diabetes Prevention Programme, “Healthier You”, has struggled to engage people under the age of 65 (NHS, 2019). Taken together, this could be as interventions and diabetes prevention programs may not address the unique barriers faced by women of reproductive age (Lim et al., 2021), such as balancing family demands, adjusting to a new role as a mother, lack of childcare and support (Dennison et al., 2019). These barriers are not exclusively within an individual’s power to overcome and change (Ioannou et al., 2024). Further understanding regarding effective intervention components, and their potential impacts on PA, is needed.

**Fig. 1:**
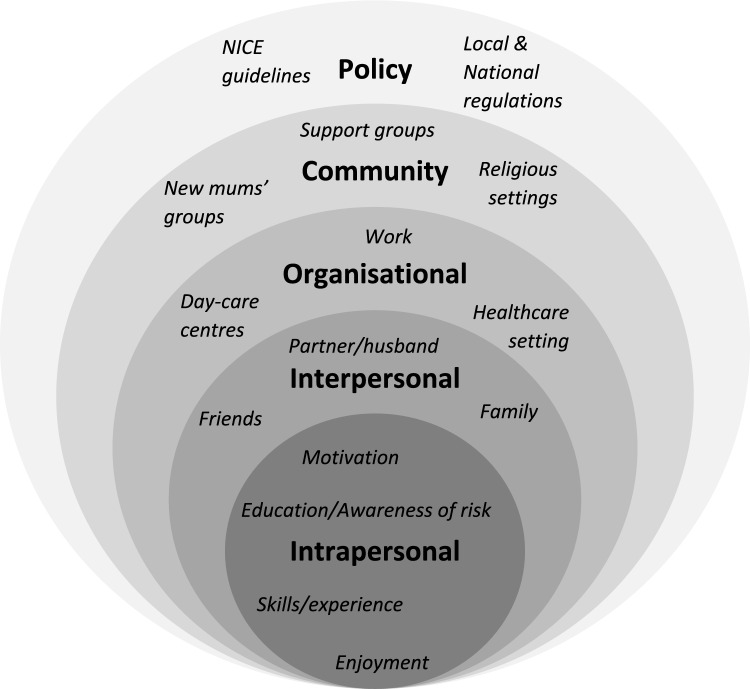
Socio-ecological model adapted to lifestyle changes in postpartum mums with previous GDM. From: McLeroy KR, Bibeau D, Steckler A, Glanz K. An ecological perspective on health promotion programs. Health Educ Q 1988, 15:351–377

An integrated system-wide approach could be more effective than single-level interventions to overcome barriers to health behaviours and improve health outcomes (Rutter et al., 2017). This is because individual behaviours do not happen in isolation, with cultural, social and other contextual factors largely determining health behaviours (McGlashan et al., 2018). The Socio-Ecological Model (SEM) focuses on the relationships between individuals and their surrounding social, physical and policy environments (Stokols, 1996). Identifying and targeting multiple levels of the SEM could therefore result in longer-term sustained behaviour change (Mcleroy et al., 1988). Figure 1 displays an adapted version of the SEM used as an a priori framework for the present study, highlighting the five levels of influence on individual behaviour.

Previous systematic reviews have examined the effectiveness of lifestyle interventions in women with previous GDM. These have evolved over the past five years, with some of the first reviews examining whether lifestyle interventions can reduce risk of T2DM in women with previous GDM (Chasan-Taber, 2015). More recently, reviews have focused on the cost-effectiveness of lifestyle interventions and the benefits and timing of lifestyle interventions (Goveia et al., 2018; Hewage et al., 2020). Only two reviews have specifically looked at intervention design. Peacock et al., (2014) highlighted that most interventions may not be translatable into real life settings. Jones et al., (2017a, 2017b) summarised knowledge and practices around tailoring multi-modal lifestyle interventions. Neither review analysed intervention components based on existing theory. Buelo et al., (2019) examined both the effectiveness of interventions and the extent to which factors influencing intervention effectiveness were addressed. Their mixed method synthesis evaluated to what extent barriers identified have been addressed in lifestyle interventions. They grouped their qualitative themes based on Dahlgren and Whitehead’s determinants of health model (Dahlgren & Whitehead, 1991), but did not analyse the intervention components according to the model.

Reviews in other topics have analysed interventions according to the SEM, and subsequent PA outcomes (Mehtälä et al., 2014). This approach has not been used before for interventions aiming to promote activity in women with previous GDM. Evaluating to what extent these interventions have incorporated a socio-ecological approach in their design, and understanding what effect specific components within each level may have on PA outcomes, can inform future intervention design in this area and subsequent policy decisions.

### Aim

The aim is to investigate the extent interventions to prevent T2DM after GDM have integrated a socio-ecological approach, and the impact on PA outcomes. The questions the review addressed included:

1. How do current PA intervention components map against different levels of the SEM?
2. How many levels of the SEM are incorporated in interventions with increases in PA?
3. Which intervention components across the SEM are commonly utilised in interventions with increases in PA?

## Methods

Methods fully comply with the PRISMA 2020 checklist (Page et al., 2021). A protocol was prepared for registration in PROSPERO (ID: CRD42021272044) but was not published elsewhere.

### Eligibility

Table 1 includes a summary of the inclusion criteria. Studies had to include women with a GDM diagnosis in the previous 5 years and have any type of PA component. Interventions initiated in pregnancy, with the aim of changing postpartum or life-long behaviours were also included. PA did not have to be the sole focus of the intervention, meaning interventions including diet or weight-loss targets were still included. If weight loss was targeted and if there were dietary components to the intervention, it was still included. To map intervention components using the SEM, any study type, e.g., protocol papers, were included.

**Table 1:** Inclusion criteria summary

Inclusion Criteria	Include	Exclude
Population	Women with a history of GDM	Women with current/previous T1DM or T2DM
Intervention	• PA (any body movement, whether purposeful exercise, whole day PA etc.) • PA plus dietary (including a dietary component to interventions)	• Breastfeeding interventions • Pharmacological interventions
Comparator	Any or none	–
Outcomes	*All review questions:* Reporting of intervention components e.g., description of details, settings where implemented etc.; and specific details and aims of the intervention itself like frequency, intensity, time, and type	*All review questions:* If there is no mention of the intervention components, activity types, details etc
	*Review Question 2 & 3:* Behavioural outcomes like PA measures (self-report weekly PA, amount of moderate-vigorous activity, activity as measured by accelerometer etc.)	*Review Question 2 & 3:* if there is no PA measurement, or measurement to see effects of intervention
Study Type	*All review questions:* Any paper reporting intervention design and components used	*All review questions:* No design or intervention components reported
	*Review Question 2 & 3:* Any study reporting primary data	*Review Question 2 & 3:* No primary data

Table provides a summary for the eligibility criteria of the present review. Each inclusion criterion was separated by the review questions being addressed. *GDM* Gestational Diabetes Mellitus; *T1DM* Type 1 Diabetes Mellitus; *T2DM* Type 2 Diabetes Mellitus; *PA* Physical Activity

### Search Strategy

A literature search was carried out in October 2022. The search was conducted in 5 databases: MEDLINE (via EBSCO), Cochrane library, Web of Science (via Clarivate analytics), CINAHL Complete (via EBSCO), and Scopus. Search terms and keywords from previous reviews of similar themes were included or modified for the purpose for the present review (Buelo et al., 2019; Goveia et al., 2018; Hewage et al., 2020). Where it was possible to include limits, results were filtered to only include publications in English language. Date limits were applied, excluding papers published before January 2000, as done by Peacock et al., (2014). A breakdown of the themes used, and search terms is displayed in Table 2.

**Table 2:** Search terms used for each analogous theme

Theme 1	“Diabetes, Gestational” [Mesh] OR “Diabetes, Pregnancy-Induced” OR “Diabetes, Pregnancy Induced” OR “Pregnancy-Induced Diabetes” OR “Pregnancy Induced Diabetes” OR “diabetes in pregnancy” OR “Gestational Diabetes” OR “Diabetes Mellitus, Gestational” OR “Gestational Diabetes Mellitus”
Population – *Women with history of GDM*	
*AND*	Exercise[Mesh] OR Exercises OR “Physical Activity” OR “Activities, Physical” OR “Activity, Physical” OR “Physical Activities” OR “Exercise, Physical” OR “Exercises, Physical” OR “Physical Exercise” OR “Physical Exercises” OR Diet[Mesh] OR Diets OR “Body Weight”[Mesh] OR “Weight, Body” OR “Weight Loss”[Mesh] OR “Loss, Weight” OR “Losses, Weight” OR “Weight Losses” OR “Weight Reduction” OR “Reduction, Weight” OR “Reductions, Weight” OR “Weight Reductions” OR “Life Style”[Mesh] OR “Life Styles” OR Lifestyle OR Lifestyles OR Education* OR family OR families OR “Web Application” OR Smartphone OR “Group Activit*” OR “Group Based” OR “Group-Based” OR Program* OR “Prevention Program*” OR Prevention
Theme 2	
Intervention – *Lifestyle changes*	
*AND*	
Theme 3	“Diabetes Mellitus, Type 2” [Mesh] OR “Type 2 Diabetes Mellitus” OR “Type 2 Diabetes” OR “Type II Diabetes” OR “Type II Diabetes Mellitus” OR “Diabetes Mellitus, Type II”
Outcome – *T2DM prevention*	

Table displays themes used for search, combined with “AND”. Within each theme, search terms were combined with “OR”. Phrases were grouped with “”. Truncation was used and depicted with asterix (*) within the table. *GDM* Gestational Diabetes Mellitus; *T2DM* Type 2 Diabetes Mellitus

### Selection Process

Screening consisted of two rounds; title and abstract followed by full-text screening (EI). At both title and abstract and full-text screening stage, a second reviewer (HH) independently screened a 10% sample of the identified papers. Provided inter-rater agreement was at least 95% and Cohen’s Kappa displayed substantial agreement, EI proceeded with data extraction. Any discrepancies were resolved via discussion. No blinding of study authors or journal title occurred.

### Data Collection Process

Data was extracted using a standardised data extraction excel sheet piloted on three papers by EI. Published papers were grouped together when they were related to a singular intervention or study. For example, where the data needed to answer the review questions spanned across a protocol and a results paper, these were grouped by the intervention name, with data items recorded as one entry and relevant information extracted from all linked publications. A maximum of two attempts were made to contact a study’s author where data was unavailable.

### Quality Assessment

The included studies were evaluated for risk of bias using the empirically grounded quality assessment tool, QualSyst, by Kmet et al.,(2004). QualSyst provides a systematic, reproducible, and quantitative means of assessing the quality of research from different study types (Kmet et al., 2004). The present study included different study types, providing valuable information to answer the research questions which would otherwise not have been considered (Clarke & Oxman, 2003; Hawker et al., 2002). Therefore, QualSyst, a more generic quality assessment tool, was suitable for assessing risk of bias in included, variable study design types. Lee et al., (2008) defined the quality of the paper based on QualSyst summary scores as strong (> 0.80), good (0.71–0.79), adequate (0.50–0.70) and limited (< 0.50). These boundaries were used in the present review to narratively synthesise the differences between findings for higher or lower quality studies (Booth et al., 2012). Studies were scored ‘N/A’, ‘2’ for ‘YES’, ‘1’ for ‘PARTIAL’ and ‘0’ for ‘NO’ on 14 different items. The total possible score was double the number of ‘N/As’ subtracted from 28. A summary score was calculated by summing the total score and dividing by the total possible score. The 14 data items scored included (extracted directly from Kmet et al., 2004):

1. Question / objective sufficiently described?
2. Study design evident and appropriate?
3. Method of subject/comparison group selection or source of information/input variables described and appropriate?
4. Subject (and comparison group, if applicable) characteristics sufficiently described?
5. If interventional and random allocation was possible, was it described?
6. If interventional and blinding of investigators was possible, was it reported?
7. If interventional and blinding of subjects was possible, was it reported?
8. Outcome and (if applicable) exposure measure(s) well defined and robust to measurement/misclassification bias? Means of assessment reported?
9. Sample size appropriate?
10. Analytic methods described/justified and appropriate?
11. Some estimate of variance is reported for the main results?
12. Controlled for confounding?
13. Results reported in sufficient detail?
14. Conclusions supported by the results?

### Synthesis Methods

An adapted SEM was used as an a priori framework to classify intervention components (Fig. 1). Each circle represents a ‘level’, and each level is labelled e.g., interpersonal. For this study, the intrapersonal level was defined as intervention components targeting psychological factors e.g., behaviour change strategies and/or educational components. The interpersonal, or social, level included components related to other individuals surrounding women who have had GDM e.g., their partners, or intervention delivery staff. The organisational level was defined as where components were targeted at or based in organisations. For example, out of healthcare settings or remotely, or the inclusion of childcare. The community level was used to represent interventions making use of community or locally based resources, while the policy level was taken to represent guidelines utilised in interventions.

PA outcomes as reported by each study were categorised as ‘U’ if no outcomes were available e.g., if the paper was a protocol paper, ‘N’ if there were no changes in PA, ‘Y’ if PA outcomes increased and ‘Y*’ if these were significant increases. These were narratively synthesised to better understand commonly utilised intervention components within and across levels of the SEM, according to PA outcomes. Interventions were labelled alphabetically. These letters were used in tables to group interventions under one label and to visually depict patterns of intervention components by SEM level.

## Results

**Fig. 2:**
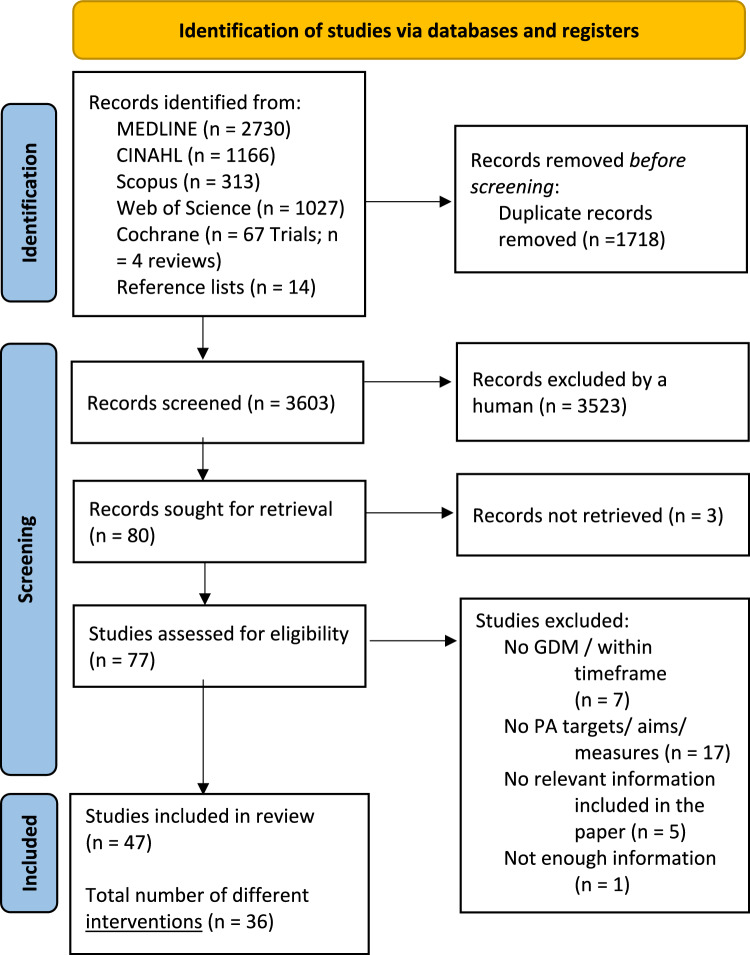
PRISMA 2020 flow diagram for systematic reviews which include searches of databases. From: Page MJ, McKenzie JE, Bossuyt PM, Boutron I, Hoffmann TC, Mulrow CD, et al. The PRISMA 2020 statement: an updated guideline for reporting systematic reviews. BMJ 2021;372:n71. https://doi.org/10.1136/bmj.n71. For more information, visit: http://www.prisma-statement.org/

After duplicates were excluded, a total of 3603 publications were retrieved from the database searches and reference lists. After screening the titles and abstracts, 77 publications were sought and further assessed for eligibility. At full-text screening, papers were excluded because they did not include relevant (n = 5), or enough information (n = 1). Some studies were also excluded due to a sole weight-loss focus, with no measures of PA behaviour change (n = 17) or because the target for intervention timing was outside of the 5-year postpartum period after GDM (n = 7). After full-text screening, 47 publications were included in the final review (comprising 36 different interventions) (Fig. 2). A summary of characteristics of included papers is displayed in Table 3.

**Table 3:** Summary of study characteristics

Intervention	Author (date)	Country	Study type	SEM levels		WL	Target	Timing	Duration	Effects on PA		Q
				#	type							
a) ADAPT-M	Lipscombe et al., (2019)	Canada	Pilot RCT	5	I, S, O, C, P	No	Both	12–24 wks PP	24 wks	Couldn’t obtain PA information	U	0.59 (A)
b) Adios diabetes	Seely et al., (2020)	USA	Pilot pre-post	3	I, S, P	Yes	Both	Within 5 yrs GDM	8 wks	Self-efficacy for PA increased significantly	Y*	0.33 (L)
c) AUS intent	Smith et al., (2014)	Australia	RCT	2	I, S	No	Both	> 6 mo PP	5 mo	Non-significant mean change in activity counts or total steps intervention vs control and no significant differences in sedentary, light, or moderate/vigorous PA time	N	0.5 (A)
d) Baby Steps	Sukumar et al., (2018)	UK (England)	Protocol (RCT)	5	I, S, O, C, P	Yes	Both	Within 60 mo GDM	12 mo	Not published yet (protocol)	U	0.64 (A)
e) Behavioural	Cheung et al., (2011)	Australia	RCT	3	I, S, P	No	PA	Within 4 yrs GDM	12 mo	At the end of the intervention, a greater but non-significant percentage of the intervention group achieved > 10,000 steps at least 5 days per week and > 150min/week MVPA vs the control	Y	0.38 (L)
f) Body Balance Beyond	Rollo et al., (2020)	Australia	Pilot RCT	2	I, S	Yes	Both	> 3 mo PP	6 mo	• Non-significant change from baseline MVPA min/week for high and control groups • No significant difference between the high and low groups	N	0.65 (A)
	Taylor et al*.,* (2022)									• No significant group-by-time effects for MVPA or exercise self-efficacy	N	0.61 (A)
g) CVD	Mukerji et al., (2015)	Canada (Ontario)	Pilot study	4	I, S, O, P	No	Both	3–6 mo PP	6 mo	Increases in exercise capacity at 3 mo compared to baseline, maintained at 6 mo versus baseline METS	Y	0.56 (A)
h) DEBI	Ferrara et al., (2011)	USA	Pilot RCT	3	I, S, P	Yes	Both	During pregnancy after diagnosis	Until 12 mo PP	Greater but non-significant increase in PA (condition difference in mean change in MVPA mins/week) at 6 wks and 7 mo	Y	0.69 (A)
i) Dulce Mothers	Philis-Tsimikas et al., (2014)	USA	Pilot Feasibility Trial	5	I, S, O, C, P	Yes	Both	Within 3 yrs after GDM	8 wks	Significantly larger percentage of participants met guidelines for aerobic activity and reported engaging in flexibility or strength training in month 3 and 6	Y*	0.68 (A)
j) Estudio PARTO	Chasan-Taber et al., (2014)	USA	Protocol (RCT)	4	I, S, O, P	Yes	Both	At 29 wks gestation	Until 12 mo PP	Versus baseline, intervention, and control: • At 6 wks significantly increased household activity • At 6 mo, significant decrease in sedentary time. Intervention also saw significant increase in MVPA • At 12 mo follow up, significant increases in MVPA and decreases in sedentary time versus baseline. Mean increase in MVPA slightly greater in intervention group	Y*	0.50 (A)
	Burkart et al., (2020)		RCT									0.73 (G)
l) Face-it	Nielsen et al., (2020)	Denmark	Protocol (RCT)	4	I, S, O, P	Yes	Both	10–14 wks PP	9 mo	Not published yet (protocol)	U	0.62 (A)
m) Families Defeating Diabetes (FDD)	McManus et al., (2018)	Canada	RCT	5	I, S, O, C, P	Yes	Both	Not specified	Not clear	• Total activity was higher at 12 vs 3 mo postpartum for all groups • Intervention group did not have higher median activity scores than control at 3 or 12 mo • Interventional male partners scored higher at both 3 mo and 12 mo than control group	Y*	0.56 (A)
n) GEM	Ferrara et al., (2014)	USA	Protocol (cluster RCT)	4	I, S, O, P	Yes	Both	6 wks PP	12 mo	• Non-significant increases in MVPA, vigorous PA and total volume of PA from during pregnancy to 6 mo postpartum for both groups • Only significant difference between intervention and control was for vigorous PA	Y*	0.61 (A)
	Ferrara et al., (2016)		Cluster RCT									0.59 (A)
p) GooD q) MomS	Nicholson et al., (2016)	USA	Pilot feasibility	4	I, S, O, P	Yes	Both	6 wks PP	26 wks	• PA decreased from baseline to 36 wks pregnancy, then decreased further at 6 wks postpartum and even further at 30 wks postpartum • Efficacy scores for PA were lower postpartum than at enrolment	N	0.58 (A)
r) HEALD-GDM	Johnson et al., (2017)	Canada	Protocol (RCT)	4	I, S, O, P	No	Both	Within 12 mo after GDM	24 wks	Not published yet (protocol)	U	0.64 (A)
s) Individualised (early)	McIntyre et al., (2012)	Australia	Pilot RCT	3	I, S, P	No	PA	6 wks PP	12 wks	• No significant difference between change in planned PA or planned walking for the intervention or control • A greater percentage of the intervention group vs control experienced a change in planned PA > 60 min/week and met PA goal	Y	0.56 (A)
t) Jewish & Bedouin	Zilberman-Kravits et al., (2018)	Israel	RCT	4	I, S, O, P	No	Both	Not specified	Not specified	• Intervention increased PA vs baseline at year 1 and 2 • At 1 and 2 years, a significantly greater percentage of the intervention group vs control performed little, moderate, and intense activity, and a lower percentage performed no PA	Y*	0.63 (A)
u) LINDA-Brazil	Schmidt et al., (2016)	Brazil	Protocol (RCT)	3	I, S, P	Yes	Both	10 wks to 2 yrs after GDM	18–60 mo	Not published yet (protocol)	U	0.40 (L)
v) LIVING	Gupta et al., (2019)	India, Bangladesh and Sri Lanka	Protocol for randomised trial	3	I, S, P	Yes	Both	6–12 mo PP	12 mo	Not published yet (protocol)	U	0.67 (A)
w) MAGDA	Shih et al., (2014)	Australia	Protocol (RCT)	4	I, S, O, P	Yes	Both	> 3 mo PP	12 mo	• Significant increase in percentage of intervention meeting PA goals (no significant difference vs control) • No significant difference in percentage of control achieving at least 30 min/day MVPA from baseline to 12 months, or between control and intervention • Significant difference between baseline and 12-month percentage of intervention group achieving at least 30 min/day MVPA, despite absolute decrease in numbers (larger percentage of drop out)	Y*	0.43 (L)
	O’Reilly et al., (2016)		RCT									0.67 (A)
y) Mediterranean	Perez-Ferre et al., (2014)	Spain	RCT	4	I, S, O, P	No	Both	7–12 wks PP	10 wks	• PA score significantly different from baseline for intervention group and significantly different between intervention and control group at follow up • PA pattern significantly improved in both groups at the end of the follow-up	Y*	0.62 (A)
z) MELINDA	Minschart et al., (2020)	Belgium	Protocol (RCT)	3	I, S, O	Yes	Both	6–16 wks PP	12 mo	Not published yet (protocol)	U	0.50 (A)
aa) MoMM-ii program	Brazeau et al., (2014)	Canada	Single-arm pilot	5	I, S, O, C, P	Yes	Both	Within 5 yrs after GDM	13 wks	• No changes in accelerometer PA • Pedometer data indicated significant improvement in steps per day achieved	Y*	0.64 (A)
	Brazeau et al., (2018)		postintervention							• Significant improvements in step counts for both women and partners • Significant increase in MVPA for women and partners (more participants doing more PA) • Partners had a conclusive 1-h reduction in self-reported daily sitting time (similar trend in women; more participants being less sedentary) • More participants perceived themselves to have better physical fitness • More participants in action phase of readiness to be active		0.68 (A)
ac) Moms in Motion	Stith et al., (2021)	USA	Protocol (RCT)	2	I, S	No	PA	25–35 days PP	190 days	Not published yet (protocol)	U	0.52 (A)
ad) MyAction	Infanti et al., (2013)	Ireland	RCT	3	I, S, C	Yes	Both	Not specified	12 wks	• Significant improvement in fitness from baseline to end of intervention • No significant difference for PA between groups • No significant intervention effect on PA levels	N	0.48 (L)
	O’Dea et al., (2015)											0.54 (A)
af) PAIGE	Holmes et al., (2018)	Ireland (Northern)	RCT	4	I, S, O, C	Yes	Both	4–6 wks PP	12 wks	• All 14 women in the intervention group were categorized as “active” (guidelines) at 6 months • Higher levels of physical functioning versus control (e.g., climb stairs easier)	Y	0.65 (A)
A) Phone-based MI	Reinhardt et al., (2012)	Australia	Pilot RCT	3	I, S, P	Yes	Both	6 wks PP	6 mo	The intervention group on average had a greater non-significant total PA (mins/day) and lower sitting time than the control group	Y	0.50 (A)
B) RADIEL	Rönö et al., (2014)	Finland	Protocol (RCT)	4	I, S, O, P	Yes	Both	6 wks PP	12 mo	• Intervention and control had PA increase • No significant difference in min/week PA between intervention and control	Y	0.56 (A)
	Huvinen et al., (2018)		RCT									0.63 (A)
D) Rural	Guo et al., (2018)	China	Protocol (RCT)	3	I, S, O	No	Both	> 6 wks PP	11 wks (booster at 3 mo)	• No significant difference in total PA (MET/wk) between groups • No significant group by time interaction effect on total PA (MET/wk)	N	0.65 (A)
	Chen et al*.,* (2022)		RCT									0.68 (A)
E) Smart mums with smart phones	Cheung et al., (2019)	Australia	Pilot RCT	4	I, S, O, P	Yes	Both	10–12 wks PP	26 wks	• Percentage of the intervention and control group meeting guidelines decreased from pregnancy/ baseline to end of intervention • Greater, non-significant weekly activity time and step count in intervention group vs control	Y	0.65 (A)
	Marschner et al*.,* (2021)		Protocol (RCT)					Birth	52 wks	Not published yet (protocol)	U	0.61 (A)
F) STAR MAMA	Athavale et al., (2016)	USA	Program Description + Case Studies	4	I, S, O, C	Yes	Both	6 wks PP	20 wks	Not published yet (protocol)	U	0.40 (L)
G) Tiajin	Hu et al., (2012)	China	RCT	2	I, S	Yes	Both	Not specified	4 wks (2 yrs total)	Intervention group more likely to increase leisure activity, have shorter sitting time and lower frequency of low occupational activity or inactive commuting vs control. After year 1, significantly larger percentage of intervention increased leisure activity, but not other activity types	Y*	0.70 (A)
	Liu et al., (2018)									Intervention group increased daily leisure PA and significantly greater percentage met PA guidelines. Overweight women more likely to increase leisure PA at month 3, 6 and 9, but less likely to achieve PA goals	Y*	0.70 (A)
N) TRIANGLE	Potzel et al*.,* (2021)	Germany	Intervention Mapping	2	I, S	Yes	Both	3–18 mo after birth	6 mo	No significant difference in PA at final visit between intervention and control group. No significant difference in change in PA from baseline to final visit in both groups	N	0.59 (A)
	Potzel et al*.,* (2022)		RCT									0.59 (A)
H) Virtual Reality Program	Kim et al., (2021)	South Korea	Quasi-experimental study	3	I, S, O	No	Both	Immediately after birth	12 wks	• Greater PA and mean differences post-baseline in experimental group at follow up vs control • Overall exercise routine in the experimental group lasted on average 20 min, 2.5 times per day	Y	0.54 (A)
J) Web-based	Kim et al., (2012)	USA	Pilot RCT	2	I, S	Yes	PA	> 6 wks but < 3 yrs post GDM	13 wks	• No significant changes in PA baseline to follow-up • • Larger, non-significant proportion of the intervention group did > 60 min/week of any PA vs control at end of intervention	Y	0.31 (L)
M) WENDY	Peacock et al., (2015)	Australia	RCT	2	I, S	Yes	Both	6 wks—2 yrs PP	3 mo	Intervention group had greater, non-significant increases in daily activity vs control at 3 months	Y	0.42 (L)

‘SEM levels’ refer to the levels of the socio-ecological model included as displayed in Fig. 1. *I* Intrapersonal; *S* social (interpersonal); *O* Organisational; *C* Community; *P* Policy; *RCT* randomized control trial; *GI* glycaemic index; *DPP* diabetes prevention program; *GDM* gestational diabetes mellitus; *RF* risk factors; *T2DM* type 2 diabetes mellitus; *PA* physical activity; *OGTT* oral glucose tolerance test; *MI* motivational interviewing; *WL* weight loss; *wks* weeks; *yrs* years; *mo* months; *PP* postpartum; *mins/week* minutes per week; Y* significant PA changes; *Y* non-significant PA changes; *N* no PA changes; *U* unpublished; *Q* Quality; *(A)* Adequate; *(L)* Limited; *(G)* Good; *N/A* Not Applicable. “Both” in targets column refers to the intervention including both PA and dietary targets/components

### Quality and Study Type

Table 4 displays study quality by study type. Twenty-four studies were RCTs and 16 were protocol studies. Most studies were “adequate” quality (n = 38), with only one study falling in the “good” quality range and the remaining limited quality (n = 8). The only “good” quality study was an RCT and saw significant PA increases (see Table 3, study (j) ‘Estudio PARTO’ by Burkart et al., (2020) for details).

**Table 4:** Summary of study types included in the review and analogous study quality

Study Type	N	Quality		
		Limited (n)	Adequate (n)	Good (n)
Protocol studies				
Protocol (RCT)	12	2	10	0
Protocol of cluster RCT	1	0	1	0
Program Description + Case Studies	2	1	1	0
Pilot studies				
Pilot RCT	8	1	7	0
Pilot Feasibility Trial	2	0	2	0
Pilot study	1	0	1	0
Single-arm pilot intervention	1	0	1	0
Pilot pre-post	1	1	0	0
Experimental studies				
RCT	16	3	12	1
Quasi-experimental study	1	0	1	0
Pre-post intervention	1	0	1	0
Cluster RCT	1	0	1	0
**Total**	**47**	**8**	**38**	**1**

*RCT* Randomised Control Trial; *N* total number of studies; *n* sub-number of studies

### Effects on PA

Table 3 highlights the effects on PA and the study type of each included paper. Of the 17 protocol papers (Chasan-Taber et al., 2014; Rönö et al., 2014; Shih et al., 2014; Ferrara et al., 2014; Athavale, et al., 2016; Schmidt et al., 2016; Johnson et al., 2017; Sukumar, et al., 2018; Guo et al., 2018; Gupta et al., 2019; Lipscombe et al., 2019; Minschart et al., 2020a; Nielsen et al., 2020; Stith et al., 2021; Marschner et al*.*, 2021; O’Reilly et al*.*, 2021; Potzel et al*.*, 2021), seven were grouped with subsequent papers reporting results. Of the interventions with published PA results, six saw no increases in PA (8 papers; (Infanti et al., 2013; Smith et al., 2014; O’Dea et al., 2015; Nicholson et al., 2016; Rollo et al., 2020; Chen et al*.*, 2022; Potzel et al*.*, 2022; Taylor et al*.*, 2022), 11 saw non-significant increases in PA (Cheung et al., 2011, 2019; Ferrara, et al., 2011; Reinhardt et al., 2012; Kim et al., 2012; McIntyre et al., 2012; Peacock et al., 2015; Mukerji et al., 2015; Holmes et al., 2018; Huvinen, et al., 2018; Kim, et al., 2021b) and 10 saw significant increases in PA (12 papers; Hu et al., 2012; Brazeau et al., 2014; Philis-Tsimikas et al., 2014; Pérez-Ferre et al., 2015; O’Reilly et al., 2016; Ferrara et al., 2016; Brazeau et al., 2018; Zilberman-Kravits et al., 2018a; Liu et al., 2018; McManus et al., 2018; Burkart et al., 2020; Seely et al., 2020).

### Levels of Influence

**Fig. 3:**
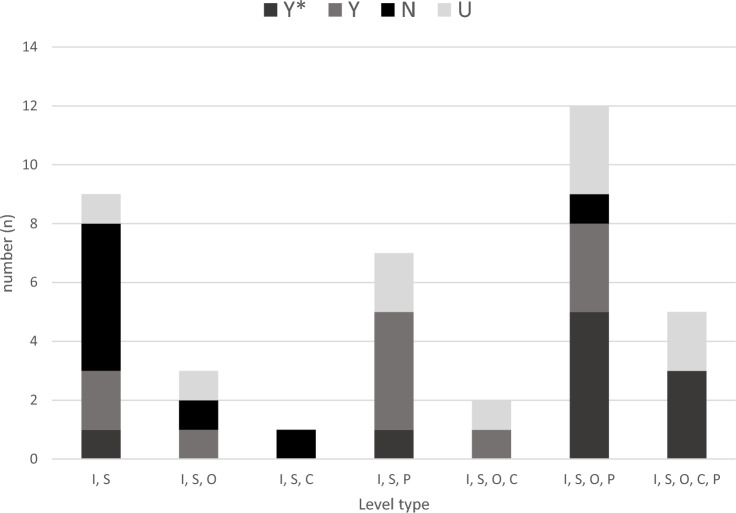
Configuration of level type by number of interventions. Key: *I* Intrapersonal; *S* social (interpersonal); *O* Organisational; *C* Community; *P* Policy; Y* significant PA changes; *Y* non-significant PA changes; *N* no PA changes; *U* unpublished

Intervention components at the intrapersonal and social levels appeared in every intervention (n = 36), with the second most commonly appearing level being Policy (n = 24). Community level components appeared the least often (n = 8), and organisation level components the second least (n = 22). Five interventions utilised all five levels of the SEM (14%), 14 had four levels (41%) and 11 had three levels (30%). More interventions with components at 4 or 5 levels of the SEM saw significant PA increases. None of the interventions had only one level of influence (Fig. 3).

### Intervention Components by Level

Table 5 summarises the intervention components according to the a priori framework used for analysis.

**Table 5:** How intervention strategies align with the adopted a-priori socio-ecological model

	Intrapersonal						Interpersonal/Social					Community		Organisational				Policy
	BCT	Edu	Ex given how	Mtr	App /web	Rdrs / msgs	HCP	Lay	Fam	Forum	Group	Comty	Local	Remt	Ex during	Childcare	Hosp	PA guidelines
**NO** PA increase (N = 6)	c, f, y, N, D	f, n, y, N, D	f	c, N	n, N	c, f, n	f, n, y, N	–	y, D	n	y	y	n, y	c, D	y, D	–	n	n
***n***	***5***	***5***	***1***	***2***	***2***	***3***	***4***	***0***	***2***	***1***	***1***	***1***	***2***	***2***	***2***	***0***	***1***	***1***
**WITH** PA increase (N = 11)	g, h, p, z, A, B, J, M	e, z, A, B, E, J, M	g, H	e, z, B, E, J, M	H	e, z, E, J, M	h, p, A, B, H, M	–	B	J	z, B, M	z, B	B	g, h, p, z, A, E	p	–	z, E, H	e, g, h, p, A, B, E
***n***	***8***	***7***	***2***	***6***	***1***	***5***	***6***	***0***	***1***	***1***	***3***	***2***	***1***	***6***	***1***	***0***	***3***	***7***
**SIGNIFICANT** PA increase (N = 10)	b, i, j, m, t, u, w, G	b, i, j, l, m, q, t, u, G	b, u	j, w	l, m, w, G	b, l, m, w	m, q, t, G, j	l, m, w	i, w	b, w	l, q, t, u, w	i	i, l, t	j, m, t, w, G	i, l, q, u, w	i, l, t, w	j, m, q, u	i, j, m, q, t, u, w
***n***	***8***	***9***	***2***	***2***	***4***	***4***	***5***	***3***	***2***	***2***	***5***	***1***	***3***	***5***	***5***	***4***	***4***	***7***
No results (N = 9)	a, d, k, r, s, v, F	a, d, k, o, r, s, v, F	a, o	d, o	d, k, v	d, r, x, F	a, d, k, v, F	d, o, s, F	d, k, o	d, k	d, o, r, s	k, F	d, F	a, o, r, s, v, F	o	a, d, o	d, k, v	a, d, k, r, s
***n***	***7***	***8***	***2***	***2***	***3***	***4***	***5***	***4***	***3***	***2***	***4***	***2***	***2***	***6***	***1***	***4***	***3***	***5***
*N* = *36*	*28*	*29*	*7*	*12*	*10*	*16*	*20*	*7*	*8*	*6*	*13*	*6*	*8*	*19*	*9*	*8*	*11*	*20*
***%***	***78***	***81***	***19***	***33***	***28***	***44***	***53***	***19***	***22***	***17***	***36***	***17***	***22***	***53***	***25***	***22***	***31***	***56***

Letters displayed refer to each of the following interventions: *a* ADAPT-M; *b* Adios diabetes; *c* AUS intent; *d* Baby Steps; *e* Behavioural; *f* Body Balance Beyond; *g* CVD; *h* DEBI; *i* Dulce Mothers; *j* Estudio PARTO; *k* Face-it; *l* Families Defeating Diabetes (FDD); *m* GEM; *n* GooDMomS; *O* HEALD-GDM; *p* Individualised (early); *q* Jewish & Bedouin; *r* LINDA-Brazil; *s* LIVING; *t* MAGDA; *u* Mediterranean; *v* MELINDA; *w* MoMM-ii program; *x* Moms in Motion; *y* MyAction; *z* PAIGE; *A* Phone-based MI; *B* RADIEL; *D* Rural; *E* Smart mums with smart phones; *F* STAR MAMA; *G* Tiajin; *N* TRIANGLE; *H* Virtual Reality Program; *J* Web-based; *M* WENDY. See Table 3 for more detailed information of each intervention

*BCT* Behaviour change strategies; *Edu* Education; *Ex* Exercises; *Mtr* Monitor; *App* Application; *Web* Web-based/websit; *Rdrs* Reminders; *Msgs* Messages; *PA* Physical Activity; *HCP* Health Care Professionals; *Lay* Lay people deliver; *Fam* Family, partner etc.; *Comty* community based; *Remt* remote; *Hosp* hospitals

Behaviour change strategies include goal setting, motivational interviewing, action plans, self-monitoring (logbook), reminders etc. Education refers to DPP content, risk perception, healthy lifestyle advice, printed materials etc. Exercises given refers to a program given or instructional/how to do exercise. Monitor refers to use of accelerometer or pedometer. App or web refers to either a program or component if the intervention including technology (website, application etc.). Reminders/messages refer to any automated messages, postcards or feedback given HCP refers to who delivered the intervention (e.g., midwife, dietitian). Family, partner etc. refers to being able or these being included in intervention. Forum refers to forums or online ways of connecting. Group refers to in-person group-based sessions. Community refers to being in community contexts or community driven. Local refers to local settings or taking place locally. Remote delivery refers to intervention delivery via phone or calls for motivation. Exercise during session refers to implementing group walks or any intervention where exercise was completed during (usually face-to-face) sessions. Childcare refers to some form of childcare or reimbursement provided. Hospitals refers to the intervention taking place, in part of fully, at the hospital in which GDM was diagnosed or cared for

#### Intrapersonal

Six different intervention components were identified at the intrapersonal level. Education was the most common component, either using diabetes prevention program content, addressing risk perception, giving healthy lifestyle advice or printed materials. Behaviour change strategies were the next most included component, referring to individualised aspects: goal setting, motivational interviewing, action plans, self-monitoring such as using a logbook and problem solving. Nearly half of the interventions also used reminders, automated messages or providing feedback. Less than one-third of the interventions gave monitors or used apps or web programmes to deliver the intervention. Approximately one-fifth of the interventions gave exercises via instructional videos or provided instruction of how to complete exercises. Patterns of PA results were similar across the intrapersonal components identified, meaning no components were identified which occurred more often in interventions seeing PA increases.

#### Social (Interpersonal)

Five different intervention components were identified under the social (interpersonal) level. The most common social component was using HCPs including exercise physiologists, dietitians, midwives to deliver the intervention. The second most common social component was using group-based sessions. All but one intervention including this component saw PA increases. Of the few interventions delivered by laypeople, three saw significant PA increases, and the rest had yet to publish results. One-fifth of the interventions allowed participants to bring their family or partner to sessions or actively included them in the interventions. The final, and least used intervention component at the social level was using forums for connecting women to each other, to ask questions or share their experiences and tips.

#### Organisational

Four different intervention components were identified at the organisational level. Remote delivery of the intervention was the most utilised intervention component, and all papers with published results reported PA increases. The next most used intervention component was being based out of hospitals where women were cared for during pregnancy. Implementing exercise during the session and providing childcare during sessions were the least used overall, yet most used in interventions with significant PA increases.

Aside from these main four components, one intervention also provided healthy food and drink at sessions and provided transportation (Guo et al., 2018). One other intervention removed the issue of cost by providing access, for example, to pool and PA services for free (Rönö et al., 2014). Finally, two interventions provided a gift card or form of monetary incentive for participating in the intervention (Lipscombe et al., 2019; McManus et al., 2018).

#### Community

Only two intervention components were identified at the community level. These components included basing interventions in local settings or involving local communities as part of the intervention. For example, the ‘MAGDA’ study and ‘Dulce mothers’ carried out sessions in community health centres and both saw significant PA increases (O’Reilly et al., 2016; Philis-Tsimikas et al., 2014; Shih et al., 2014). The ‘Families Defeating Diabetes’ intervention had walking groups taking place in local malls and also saw significant increases in PA (McManus et al., 2018).

#### Policy

Fifty-six percent of the interventions implemented PA guidelines (n = 20). This included country-specific guidelines, such as the Chief Medical Officer’s guidelines in the UK, or more generally the WHO PA guidelines (Davies et al., 2019; WHO, 2016). This was the only component stated at a policy level. Use of PA guidelines was similarly spread across different PA results.

## Discussion

The aim of the present review was to: (a) map PA intervention components using the SEM, (b) understand how many levels and (c) determine what intervention components across the SEM are commonly utilised in interventions seeing PA increases. Overall, significant PA increases mostly occurred when four or all five levels of the SEM were utilised. Intervention components which had more increases in PA were remote delivery of the intervention, providing childcare, and having group-based sessions.

In addition to the 16 protocol papers, expanding the inclusion criteria for any study type resulted in an additional eight papers included in the present review, mainly as pilot, feasibility, or pre-post studies. Mixed PA outcomes were observed from these eight papers, therefore, it was not the case that non-RCTs were more likely to show meaningful PA increases. It is possible that, due to standards for publishing RCTs, these tend to include more explicit information regarding study design (Kmet et al., 2004; Schulz et al., 2010). However, QualSyst’s performance as a quality assessment tool seemed evenly spread over the different study types, therefore it is likely that quality was adequately assessed, and that studies generally were not reported well.

All interventions included both the intrapersonal and social levels. These levels also included the greatest variety of intervention components. This is important, as these levels of influence are theorised as having the strongest influences on an individual (Kilanowski, 2017). Use of behaviour change strategies to reduce T2DM risk after GDM has also been determined as important, at the very least for reducing energy intake (Lim et al., 2020). While interventions should include the intrapersonal level due to the influences on individual behaviour, it is likely that ability to increase PA is constrained by wider factors across the other levels. This is evidenced in the present review as significant PA increases occurred mostly when four or five levels of the SEM were utilised. Additionally, the present review identified that intervention components used across the intrapersonal level, including use of behaviour change techniques, showed mixed PA outcomes, with components across other levels showing greater variation and more definitive. Therefore, while the intrapersonal level matters, wider levels of the SEM may act as constraints for increasing activity in women with previous GDM and therefore need to be included in interventions.

Despite the number of and type of level potentially impacting PA outcomes, findings of the present review indicate that intervention components within each level are also important. More specifically, distinctive patterns across intervention components from the social and organisational levels were seen. For example, providing childcare (organisational) was a key component that appeared most in interventions seeing significant PA increases, and did not appear in interventions with no or non-significant PA increases. This result is in agreement with literature which has identified childcare (or lack of), as a barrier to participation, given women’s identified “role as a mother” (Dennison et al., 2019a). In terms of the SEM, childcare as a barrier is not wholly within an individual’s capability to overcome (Ioannou et al., 2024). It is a structural barrier, which from a practical perspective, to overcome, would need to be addressed by the non-intrapersonal levels of the SEM (Ioannou et al., 2023). To increase activity, it is therefore important that interventions targeting women after GDM not only target behaviour change strategies, but also address barriers at either the organisational or community level, for example, by addressing social norms around the role of a mother, and/or providing childcare.

Group-based (social) and remote delivery (organisational) were also most seen in interventions with significant and non-significant PA increases. This may seem conflicting, however, a blended approach could improve PA outcomes in future interventions. Tang et al., (2015) highlighted that PA done at home could better engage women after GDM, as lack of time and flexibility were key barriers. However, group-based sessions could be effective for managing chronic conditions (Harden et al., 2015). Specifically, women after GDM value connections made with other women who have shared a similar experience (Kelly-Whyte et al., 2021). While it could be expected that forums would provide a similar sort of comfort, the present review did not find this to be a particularly beneficial intervention component. In part, this could be because forums are less personal, and could not be providing the type of social support women with previous GDM are looking for. A recent study by Dennison et al., (2022) highlighted that women after GDM want more support, including connecting and meeting with other mums who have had GDM. Therefore, connecting women with previous GDM e.g., through group-based sessions, where there is flexibility to incorporate PA at home and in own time, could be useful to improve PA outcomes in interventions trying to reduce T2DM risk.

### Limitations

The present review categorised intervention components based on where they sit within a system, however, SEM levels refer to systems changes (Mcleroy et al., 1988). For example, using HCPs to deliver the intervention was categorised at the social level. While this is a social interaction, the interventions were not actively targeting HCP behaviour, knowledge, or attitudes to help or benefit women. While the present review used the SEM to map intervention components and design, interventions should focus on at least two different levels of influence (Stokols, 1996). Using the example above, this could involve targeting beliefs HCPs hold that may be unhelpful, to enable them to most effectively provide the support that women after GDM have indicated they would like to receive (Dennison et al., 2022).

Another limitation of the present review was how PA outcomes were quantified and interpreted. PA targets and measures in the identified interventions were heterogeneous. To accommodate for this, and enable meaningful synthesis, an intervention was categorised as having successful PA outcomes, based on whatever PA outcomes were used within the individual study. However, it is important to note that reporting of PA outcomes was greatly varied. How interventions themselves classified significance also varied. To accommodate for this, this review considered and looked for patterns across interventions seeing changes in PA, whether these were or were not significant. While this method was useful for synthesising and understanding the results of the review, it is limited. However, results of the review were consistent with other literature discussed above, providing a degree of confidence.

## Conclusions

While interventions to prevent T2DM after GDM do incorporate multiple levels of the SEM, those which included components at the organisational levels, targeting structural barriers like providing childcare, had a greater number of significant PA increases. Future interventions targeting this population should, at the very least, address childcare barriers in their intervention design. They should also consider how to encourage social support between women who have had GDM, for example, through group-based sessions, and consider how the offer of remote delivery can provide increased flexibility for participation.

## Data Availability

All data used was obtained from published articles and was not generated.

## References

[CR1] Athavale, P., Thomas, M., Delgadillo-Duenas, A. T., Leong, K., Najmabadi, A., Harleman, E., Rios, C., Quan, J., Soria, C., & Handley, M. A. (2016). Linking high risk postpartum women with a technology enabled health coaching program to reduce diabetes risk and improve wellbeing: Program description, case studies, and recommendations for community health coaching programs. *Journal of Diabetes Research,**2016*, 4353956. 10.1155/2016/4353956 27830157 10.1155/2016/4353956 PMC5088315

[CR2] Ayman, G., Strachan, J. A., McLennan, N., Malouf, R., Lowe-Zinola, J., Magdi, F., Roberts, N., Alderdice, F., Berneantu, I., Breslin, N., Byrne, C., Carnell, S., Churchill, D., Grisoni, J., Hirst, J. E., Morris, A., Murphy, H. R., O’Brien, J., Schmutz, C., & Knight, M. (2021). The top ten research priorities in diabetes and pregnancy according to women, support networks and healthcare professionals. *Diabetic Medicine,**38*(8), e14588. 10.1111/dme.14588 33949704 10.1111/dme.14588 PMC8359941

[CR3] Bao, W., Tobias, D. K., Bowers, K., Chavarro, J., Vaag, A., Grunnet, L. G., Strøm, M., Mills, J., Liu, A., Kiely, M., & Zhang, C. (2014). Physical activity and sedentary behaviors associated with risk of progression from gestational diabetes mellitus to type 2 diabetes mellitus: A prospective cohort study. *JAMA Internal Medicine,**174*(7), 1047–1055. 10.1001/jamainternmed.2014.1795 24841449 10.1001/jamainternmed.2014.1795 PMC4209161

[CR4] Bentley-Lewis, R., Levkoff, S., Stuebe, A., & Seely, E. W. (2008). Gestational diabetes mellitus: Postpartum opportunities for the diagnosis and prevention of type 2 diabetes mellitus. *Nature Clinical Practice Endocrinology & Metabolism,**4*(10), 552–558. 10.1038/ncpendmet0965 10.1038/ncpendmet0965 PMC4428574 18779843

[CR5] Booth, A., Sutton, A., & Papaioannou, D. (2012). Systematic approaches to a successful literature review. *Journal of the Canadian Health Libraries Association/journal De L’association Des Bibliothèques De La Santé Du Canada,**34*(1), 009. 10.5596/c13-009 10.5596/c13-009

[CR6] Brazeau, A. S., Leong, A., Meltzer, S. J., Cruz, R., DaCosta, D., Hendrickson-Nelson, M., Joseph, L., Dasgupta, K., Bacon, S., Stotland, S., Chetty, V. T., Gougeon, R., Garfield, N., & Majdan, A. (2014). Group-based activities with on-site childcare and online support improve glucose tolerance in women within 5 years of gestational diabetes pregnancy. *Cardiovascular Diabetology,**13*(1), 104. 10.1186/1475-2840-13-104 24981579 10.1186/1475-2840-13-104 PMC4227099

[CR7] Brazeau, A. S., Meltzer, S. J., Pace, R., Garfield, N., Godbout, A., Meissner, L., Rahme, E., Da Costa, D., & Dasgupta, K. (2018). Health behaviour changes in partners of women with recent gestational diabetes: A phase IIa trial. *BMC Public Health,**18*(1), 575. 10.1186/S12889-018-5490-X 29716559 10.1186/S12889-018-5490-X PMC5930949

[CR8] Buelo, A. K., Kirk, A., Lindsay, R. S., & Jepson, R. G. (2019). Exploring the effectiveness of physical activity interventions in women with previous gestational diabetes: A systematic review of quantitative and qualitative studies. *Preventive Medicine Reports,**14*, 100877. 10.1016/j.pmedr.2019.100877 31110933 10.1016/j.pmedr.2019.100877 PMC6510702

[CR9] Burkart, S., Marcus, B. H., Pekow, P., Rosal, M. C., Manson, J. E., Braun, B., & Chasan-Taber, L. (2020). The impact of a randomized controlled trial of a lifestyle intervention on postpartum physical activity among at-risk hispanic women: Estudio PARTO. *PLoS ONE,**15*(7), e0236408. 10.1371/JOURNAL.PONE.0236408 32706812 10.1371/JOURNAL.PONE.0236408 PMC7380594

[CR10] Chasan-Taber, L. (2015). Lifestyle interventions to reduce risk of diabetes among women with prior gestational diabetes mellitus. *Best Practice and Research: Clinical Obstetrics and Gynaecology,**29*(1), 110–122. 10.1016/j.bpobgyn.2014.04.019 25220104 10.1016/j.bpobgyn.2014.04.019 PMC4282816

[CR11] Chasan-Taber, L., Marcus, B. H., Rosal, M. C., Tucker, K. L., Hartman, S. J., Pekow, P., Braun, B., Moore Simas, T. A., Solomon, C. G., Manson, J. E., & Markenson, G. (2014). Estudio parto: Postpartum diabetes prevention program for hispanic women with abnormal glucose tolerance in pregnancy: A randomised controlled trial – study protocol. *BMC Pregnancy and Childbirth,**14*(1), 1–8. 10.1186/1471-2393-14-100 24606590 10.1186/1471-2393-14-100 PMC3975296

[CR12] Cheung, N. W., Smith, B. J., Van Der Ploeg, H. P., Cinnadaio, N., & Bauman, A. (2011). A pilot structured behavioural intervention trial to increase physical activity among women with recent gestational diabetes. *Diabetes Research and Clinical Practice,**92*, e27–e29. 10.1016/j.diabres.2011.01.013 21316788 10.1016/j.diabres.2011.01.013

[CR13] Cheung, N. W., Blumenthal, C., Smith, B. J., Hogan, R., Thiagalingam, A., Redfern, J., Barry, T., Cinnadaio, N., & Chow, C. K. (2019). A pilot randomised controlled trial of a text messaging intervention with customisation using linked data from wireless wearable activity monitors to improve risk factors following gestational diabetes. *Nutrients,**11*(3), 590. 10.3390/NU11030590 30862052 10.3390/NU11030590 PMC6470941

[CR14] Clarke, M., & Oxman, A. D. (2003). Cochrane reviewer’s handbook 4.2.0. In *Cochrane Library, Issue 2*.

[CR15] Dahlgren, G., & Whitehead, M. (1991). *Policies and strategies to promote social equity in health*. Stockholm, Sweden: Institute for Futures Studies.

[CR16] Davies, D. S. C., Atherton, F., McBride, M., & Calderwood, C. (2019). UK chief medical officers’ physical activity guidelines. *Department of Health and Social Care*, *September*, 1–65. https://www.gov.uk/government/publications/physical-activity-guidelines-uk-chief-medical-officers-report

[CR17] Dennison, R. A., Ward, R. J., Griffin, S. J., & Usher-Smith, J. A. (2019). Women’s views on lifestyle changes to reduce the risk of developing type 2 diabetes after gestational diabetes: A systematic review, qualitative synthesis and recommendations for practice. *Diabetic Medicine,**36*(6), 702–717. 10.1111/DME.13926 30723968 10.1111/DME.13926 PMC6563496

[CR18] Dennison, R. A., Griffin, S. J., Usher-Smith, J. A., Fox, R. A., Aiken, C. E., & Meek, C. L. (2022). “Post-GDM support would be really good for mothers”: A qualitative interview study exploring how to support a healthy diet and physical activity after gestational diabetes. *PLoS ONE,**17*(1), e0262852. 10.1371/JOURNAL.PONE.0262852 35061856 10.1371/JOURNAL.PONE.0262852 PMC8782419

[CR19] Ferrara, A., Hedderson, M. M., Albright, C. L., Ehrlich, S. F., Jr Quesenberry, C. P., Peng, T., Feng, J., Ching, J., & Crites, Y. (2011). A pregnancy and postpartum lifestyle intervention in women with gestational diabetes mellitus reduces diabetes risk factors: A feasibility randomized control trial. *Diabetes Care,**34*(7), 1519–1525. 10.2337/dc10-2221 21540430 10.2337/dc10-2221 PMC3120183

[CR20] Ferrara, A., Hedderson, M. M., Albright, C. L., Brown, S. D., Ehrlich, S. F., Caan, B. J., Sternfeld, B., Gordon, N. P., Schmittdiel, J. A., Gunderson, E. P., Mevi, A. A., Tsai, A.-L., Ching, J., Crites, Y., & Jr Quesenberry, C. P. (2014). A pragmatic cluster randomized clinical trial of diabetes prevention strategies for women with gestational diabetes: Design and rationale of the gestational diabetes’ effects on moms (GEM) study. *BMC Pregnancy and Childbirth,**14*(1), 21. 10.1186/1471-2393-14-21 24423410 10.1186/1471-2393-14-21 PMC3897959

[CR21] Ferrara, A., Hedderson, M. M., Brown, S. D., Albright, C. L., Ehrlich, S. F., Tsai, A.-L., Caan, B. J., Sternfeld, B., Gordon, N. P., Schmittdiel, J. A., Gunderson, E. P., Mevi, A. A., Herman, W. H., Ching, J., Crites, Y., & Jr Quesenberry, C. P. (2016). The comparative effectiveness of diabetes prevention strategies to reduce postpartum weight retention in women with gestational diabetes mellitus: The gestational diabetes’ effects on moms (GEM) cluster randomized controlled trial. *Diabetes Care,**39*(1), 65. 10.2337/DC15-1254 26657945 10.2337/DC15-1254 PMC4686847

[CR22] Goveia, P., Cañon-Montañez, W., de Paula Santos, D., Lopes, G. W., Ma, R. C. W., Duncan, B. B., Ziegelman, P. K., & Schmidt, M. I. (2018). Lifestyle intervention for the prevention of diabetes in women with previous gestational diabetes mellitus: A systematic review and meta-analysis. *Frontiers in Endocrinology,**9*, 583. 10.3389/fendo.2018.00583 30344509 10.3389/fendo.2018.00583 PMC6182069

[CR23] Guo, J., Tang, Y., Wiley, J., Whittemore, R., & Chen, J.-L. (2018). Effectiveness of a diabetes prevention program for rural women with prior gestational diabetes mellitus: Study protocol of a multi-site randomized clinical trial. *BMC Public Health,**18*(1), 809. 10.1186/s12889-018-5725-x 29954367 10.1186/s12889-018-5725-x PMC6022415

[CR24] Gupta, Y., Kapoor, D., Josyula, L. K., Praveen, D., Naheed, A., Desai, A. K., Pathmeswaran, A., de Silva, H. A., Lombard, C. B., Alam, D. S., Prabhakaran, D., Teede, H. J., Billot, L., Bhatla, N., Joshi, R., Zoungas, S., Jan, S., Patel, A., & Tandon, N. (2019). A lifestyle intervention programme for the prevention of type 2 diabetes mellitus among South Asian women with gestational diabetes mellitus [LIVING study]: Protocol for a randomized trial. *Diabetic Medicine,**36*(2), 243–251. 10.1111/DME.13850 30368898 10.1111/DME.13850

[CR25] Harden, S. M., McEwan, D., Sylvester, B. D., Kaulius, M., Ruissen, G., Burke, S. M., Estabrooks, P. A., & Beauchamp, M. R. (2015). Understanding for whom, under what conditions, and how group-based physical activity interventions are successful: A realist review Health behavior, health promotion and society. *BMC Public Health,**15*(1), 958. 10.1186/s12889-015-2270-8 26404722 10.1186/s12889-015-2270-8 PMC4582831

[CR26] Hawker, S., Payne, S., Kerr, C., Hardey, M., & Powell, J. (2002). Appraising the evidence: Reviewing disparate data systematically. *Qualitative Health Research,**12*(9), 1284–1299. 10.1177/1049732302238251 12448672 10.1177/1049732302238251

[CR27] Hewage, S. S., Wu, S., Neelakantan, N., & Yoong, J. (2020). Systematic review of effectiveness and cost-effectiveness of lifestyle interventions to improve clinical diabetes outcome measures in women with a history of GDM. *Clinical Nutrition ESPEN,**35*, 20–29. 10.1016/j.clnesp.2019.10.011 31987117 10.1016/j.clnesp.2019.10.011

[CR28] Holmes, V. A., Draffin, C. R., Patterson, C. C., Francis, L., Irwin, J., McConnell, M., Farrell, B., Brennan, S. F., McSorley, O., Wotherspoon, A. C., Davies, M., & McCance, D. R. (2018). Postnatal lifestyle intervention for overweight women with previous gestational diabetes: A randomized controlled trial. *The Journal of Clinical Endocrinology and Metabolism,**103*(7), 2478–2487. 10.1210/jc.2017-02654 29762737 10.1210/jc.2017-02654

[CR29] Hu, G., Tian, H., Zhang, F., Liu, H., Zhang, C., Zhang, S., Wang, L., Liu, G., Yu, Z., Yang, X., Qi, L., Zhang, C., Wang, H., Li, M., Leng, J., Li, Y., Dong, L., & Tuomilehto, J. (2012). Tianjin gestational diabetes mellitus prevention program: Study design, methods, and 1-year interim report on the feasibility of lifestyle intervention program. *Diabetes Research and Clinical Practice,**98*(3), 508–517. 10.1016/j.diabres.2012.09.015 23010556 10.1016/j.diabres.2012.09.015

[CR30] Huvinen, E., Koivusalo, S. B., Meinilä, J., Valkama, A., Tiitinen, A., RöNö, K., Stach-Lempinen, B., & Eriksson, J. G. (2018). Effects of a lifestyle intervention during pregnancy and first postpartum year: Findings from the RADIEL study. *Journal of Clinical Endocrinology & Metabolism,**103*(4), 1669–1677. 10.1210/jc.2017-02477 29409025 10.1210/jc.2017-02477

[CR31] Infanti, J. J., Dunne, F. P., O’Dea, A., Gillespie, P., Gibson, I., Glynn, L. G., Noctor, E., Newell, J., & McGuire, B. E. (2013). An evaluation of Croí MyAction community lifestyle modification programme compared to standard care to reduce progression to diabetes/pre-diabetes in women with prior gestational diabetes mellitus (GDM): Study protocol for a randomised controlled trial. *Trials,**14*, 121. 10.1186/1745-6215-14-121 23782471 10.1186/1745-6215-14-121 PMC3747856

[CR32] Ioannou, E., Humphreys, H., Homer, C., & Purvis, A. (2023). Systematic review and thematic synthesis of the barriers and facilitators to physical activity for women after gestational diabetes: A socio-ecological approach. *British Journal of Diabetes,**23*(1), 2–13. 10.15277/bjd.2023.413 10.15277/bjd.2023.413

[CR33] Ioannou, E., Humphreys, H., Homer, C., & Purvis, A. (2024). Beyond the individual: Socio-ecological factors impacting activity after gestational diabetes mellitus. *Diabetic Medicine,**00*, e15286. 10.1111/DME.15286 10.1111/DME.15286 38291570

[CR34] Johnson, S. T., Mladenovic, A. B., Mathe, N., Davenport, M. H., Butalia, S., Qiu, W., & Johnson, J. A. (2017). Healthy eating and active living after gestational diabetes mellitus (HEALD-GDM): Rationale, design, and proposed evaluation of a randomized controlled trial. *Contemporary Clinical Trials,**61*, 23–28. 10.1016/J.CCT.2017.07.008 28700892 10.1016/J.CCT.2017.07.008

[CR35] Jones, E., Fraley, H., & Mazzawi, J. (2017a). Appreciating recent motherhood and culture: A Systematic review of multimodal postpartum lifestyle interventions to reduce diabetes risk in women with prior gestational diabetes. *Maternal & Child Health Journal,**21*(1), 45–57. 10.1007/s10995-016-2092-z 27435732 10.1007/s10995-016-2092-z

[CR36] Jones, E. J., Fraley, H. E., & Mazzawi, J. (2017b). Appreciating recent motherhood and culture: A systematic review of multimodal postpartum lifestyle interventions to reduce diabetes risk in women with prior gestational diabetes. *Maternal and Child Health Journal*. 10.1007/s10995-016-2092-z 27435732 10.1007/s10995-016-2092-z

[CR37] Kelly-Whyte, N., McNulty, C., & O’Reilly, S. (2021). Perspectives on mHealth interventions during and after gestational diabetes. *Current Developments in Nutrition,**5*(2), 768–768. 10.1093/CDN/NZAB046_065 10.1093/CDN/NZAB046_065

[CR38] Kilanowski, J. F. (2017). Breadth of the socio-ecological model. *Journal of Agromedicine,**22*(4), 295–297. 10.1080/1059924X.2017.1358971 28742433 10.1080/1059924X.2017.1358971

[CR39] Kim, C., Draska, M., Hess, M. L., Wilson, E. J., & Richardson, C. R. (2012). A web-based pedometer program in women with recent histories of gestational diabetes. *Diabetic Medicine : A Journal of the British Diabetic Association,**29*(2), 278. 10.1111/J.1464-5491.2011.03415.X 21838764 10.1111/J.1464-5491.2011.03415.X PMC4139030

[CR40] Kim, S.-H., Kim, H. J., & Shin, G. (2021). Self-management mobile virtual reality program for women with gestational diabetes. *International Journal of Environmental Research and Public Health,**18*(4), 1–12. 10.3390/IJERPH18041539 10.3390/IJERPH18041539 PMC7915744 33562853

[CR41] Kmet, L. M., Lee, R. C., & Cook, L. S. (2004). Standard quality assessment criteria for evaluating primary research papers from a variety of fields. *In HTA Initiative*. 10.5858/arpa.2020-0217-sa 10.5858/arpa.2020-0217-sa

[CR42] Knowler, W., Barrett-Connor, E., Fowler, S., Hamman, R., Lachin, J., Walker, E., & Nathan, D. (2002). Reduction in the incidence of type 2 diabetes with lifestyle intervention or metformin. *New England Journal of Medicine,**346*(6), 393–403. 10.1056/NEJMoa012512 11832527 10.1056/NEJMoa012512 PMC1370926

[CR43] Lee, L., Packer, T. L., Tang, S. H., & Girdler, S. (2008). Self-management education programs for age-related macular degeneration: A systematic review. *Australasian Journal on Ageing,**27*(4), 170–176. 10.1111/J.1741-6612.2008.00298.X 19032617 10.1111/J.1741-6612.2008.00298.X

[CR44] Lim, S., Hill, B., Pirotta, S., O’reilly, S., & Moran, L. (2020). What are the most effective behavioural strategies in changing postpartum women’s physical activity and healthy eating behaviours? A systematic review and meta-analysis. *Journal of Clinical Medicine,**9*(1), 237. 10.3390/JCM9010237 31963150 10.3390/JCM9010237 PMC7019954

[CR45] Lipscombe, L. L., Delos-Reyes, F., Glenn, A. J., de Sequeira, S., Liang, X., Grant, S., Thorpe, K. E., & Price, J. A. D. (2019). The avoiding diabetes after pregnancy trial in moms program: Feasibility of a diabetes prevention program for women with recent gestational diabetes mellitus. *Canadian Journal of Diabetes,**43*(8), 613–620. 10.1016/j.jcjd.2019.08.019 31669188 10.1016/j.jcjd.2019.08.019

[CR46] Liu, H., Wang, L., Zhang, S., Leng, J., Li, N., Li, W., Wang, J., Tian, H., Qi, L., Yang, X., Yu, Z., Tuomilehto, J., & Hu, G. (2018). One-year weight losses in the tianjin gestational diabetes mellitus prevention programme: A randomized clinical trial. *Diabetes, Obesity & Metabolism,**20*(5), 1246–1255. 10.1111/dom.13225 10.1111/dom.13225 PMC5899932 29360237

[CR47] McGlashan, J., Hayward, J., Brown, A., Owen, B., Millar, L., Johnstone, M., Creighton, D., & Allender, S. (2018). Comparing complex perspectives on obesity drivers: Action-driven communities and evidence-oriented experts. *Obesity Science & Practice,**4*(6), 575–581. 10.1002/osp4.306 30574350 10.1002/osp4.306 PMC6298210

[CR48] McIntyre, H. D., Peacock, A., Miller, Y. D., Koh, D., & Marshall, A. L. (2012). Pilot study of an individualised early postpartum intervention to increase physical activity in women with previous gestational diabetes. *International Journal of Endocrinology.,**2012*, 892019. 10.1155/2012/892019 22548057 10.1155/2012/892019 PMC3324899

[CR49] Mcleroy, K. R., Bibeau, D., Steckler, A., & Glanz, K. (1988). An ecological perspective on health promotion programs. *Health Education & Behavior,**15*(4), 351–377. 10.1177/109019818801500401 10.1177/109019818801500401 3068205

[CR50] McManus, R., Miller, D., Mottola, M., Giroux, I., & Donovan, L. (2018). Translating healthy living messages to postpartum women and their partners after gestational diabetes (GDM): Body habitus, A1C, lifestyle habits, and program engagement results from the families defeating diabetes (FDD) randomized trial. *American Journal of Health Promotion,**32*(6), 1438–1446. 10.1177/0890117117738210 29108443 10.1177/0890117117738210

[CR51] Mehtälä, M. A. K., Sääkslahti, A. K., Inkinen, M. E., & Poskiparta, M. E. H. (2014). A socio-ecological approach to physical activity interventions in childcare: A systematic review. *In International Journal of Behavioral Nutrition and Physical Activity,**11*(1), 22. 10.1186/1479-5868-11-22 10.1186/1479-5868-11-22 PMC3936868 24559188

[CR52] Metzger, B. E. (2010). International association of diabetes and pregnancy study groups recommendations on the diagnosis and classification of hyperglycemia in pregnancy. *Diabetes Care,**33*(3), 676–682. 10.2337/dc09-1848 20190296 10.2337/dc09-1848 PMC2827530

[CR53] Minschart, C., Maes, T., De Block, C., Van Pottelbergh, I., Myngheer, N., Abrams, P., Vinck, W., Leuridan, L., Mathieu, C., Billen, J., Matthys, C., Weyn, B., Laenen, A., Bogaerts, A., & Benhalima, K. (2020). Mobile-based lifestyle intervention in women with glucose intolerance after gestational diabetes mellitus (MELINDA), a multicenter randomized controlled trial: Methodology and design. *Journal of Clinical Medicine,**9*(8), 1–14. 10.3390/JCM9082635 10.3390/JCM9082635 PMC7465345 32823771

[CR54] Mukerji, G., McTavish, S., Glenn, A., Delos-Reyes, F., Price, J., Wu, W., Harvey, P., & Lipscombe, L. L. (2015). An innovative home-based cardiovascular lifestyle prevention program for women with recent gestational diabetes: A pilot feasibility study. *Canadian Journal of Diabetes,**39*, 445–450. 10.1016/j.jcjd.2015.08.002 26482886 10.1016/j.jcjd.2015.08.002

[CR55] NHS. (2019). *NHS England » NHS Diabetes Prevention Programme (NHS DPP)*. https://www.england.nhs.uk/ltphimenu/diabetes-prevention/nhs-diabetes-prevention-programme-nhs-dpp/

[CR56] NICE. (2020). *Diabetes in pregnancy overview – NICE Pathways*. https://pathways.nice.org.uk/pathways/diabetes-in-pregnancy/diabetes-in-pregnancy-overview#content=view-node%3Anodes-postnatal-care-for-women-who-were-diagnosed-with-gestational-diabetes

[CR57] Nicholson, W., Beckham, A., Hatley, K., Diamond, M., Johnson, L., Green, S., & Tate, D. (2016). The gestational diabetes management system (GooDMomS): Development, feasibility and lessons learned from a patient-informed, web-based pregnancy and postpartum lifestyle intervention. *BMC Pregnancy and Childbirth,**16*(1), 277. 10.1186/S12884-016-1064-Z 27654119 10.1186/S12884-016-1064-Z PMC5031324

[CR58] Nielsen, K. K., Dahl-Petersen, I. K., Jensen, D. M., Ovesen, P., Damm, P., Jensen, N. H., Thøgersen, M., Timm, A., Hillersdal, L., Kampmann, U., Vinter, C. A., Mathiesen, E. R., & Maindal, H. T. (2020). Protocol for a randomised controlled trial of a co-produced, complex, health promotion intervention for women with prior gestational diabetes and their families: The face-it study. *Trials,**21*(1), 146. 10.1186/s13063-020-4062-4 32033613 10.1186/s13063-020-4062-4 PMC7006376

[CR59] O’Dea, A., Tierney, M., McGuire, B. E., Newell, J., Glynn, L. G., Gibson, I., Noctor, E., Danyliv, A., Connolly, S. B., & Dunne, F. P. (2015). Can the onset of type 2 diabetes be delayed by a group-based lifestyle intervention in women with prediabetes following gestational diabetes mellitus (GDM)? Findings from a randomized control mixed methods trial. *Journal of Diabetes Research,**2015*, 798460. 10.1155/2015/798460 26347894 10.1155/2015/798460 PMC4546980

[CR60] O’Reilly, S. L., Dunbar, J. A., Versace, V., Janus, E., Best, J. D., Carter, R., Oats, J. J. N., Skinner, T., Ackland, M., Phillips, P. A., Ebeling, P. R., Reynolds, J., Shih, S. T. F., Hagger, V., Coates, M., & Wildey, C. (2016). Mothers after gestational diabetes in Australia (MAGDA): A randomised controlled trial of a postnatal diabetes prevention program. *PLOS Medicine,**13*(7), e1002092. 10.1371/JOURNAL.PMED.1002092 27459502 10.1371/JOURNAL.PMED.1002092 PMC4961439

[CR61] Page, M. J., McKenzie, J. E., Bossuyt, P. M., Boutron, I., Hoffmann, T. C., Mulrow, C. D., Shamseer, L., Tetzlaff, J. M., Akl, E. A., Brennan, S. E., Chou, R., Glanville, J., Grimshaw, J. M., Hróbjartsson, A., Lalu, M. M., Li, T., Loder, E. W., Mayo-Wilson, E., McDonald, S., & Moher, D. (2021). The PRISMA 2020 statement: An updated guideline for reporting systematic reviews. *BMJ,**372*, n71. 10.1136/BMJ.N71 33782057 10.1136/BMJ.N71 PMC8005924

[CR62] Pan, X. R., Li, G. W., Hu, Y. H., Wang, J. X., Yang, W. Y., An, Z. X., Hu, Z. X., Lin, J., Xiao, J. Z., Cao, H. B., Liu, P. A., Jiang, X. G., Jiang, Y. Y., Wang, J. P., Zheng, H., Zhang, H., Bennett, P. H., & Howard, B. V. (1997). Effects of diet and exercise in preventing NIDDM in people with impaired glucose tolerance: The da qing IGT and diabetes study. *Diabetes Care,**20*(4), 537–544. 10.2337/diacare.20.4.537 9096977 10.2337/diacare.20.4.537

[CR63] Peacock, A. S., Bogossian, F., McIntyre, H. D., & Wilkinson, S. (2014). A review of interventions to prevent type 2 diabetes after gestational diabetes. *Women and Birth,**27*(4), e7–e15. 10.1016/j.wombi.2014.09.002 25262356 10.1016/j.wombi.2014.09.002

[CR64] Peacock, A. S., Bogossian, F. E., Wilkinson, S. A., Gibbons, K. S., Kim, C., & McIntyre, H. D. (2015). A randomised controlled trial to delay or prevent type 2 diabetes after gestational diabetes: Walking for exercise and nutrition to prevent diabetes for you. *International Journal of Endocrinology,**2015*, 423717. 10.1155/2015/423717 26089886 10.1155/2015/423717 PMC4452189

[CR65] Perez-Ferre, N., Del Valle, L., Torrej, M. J. E., Barca, I., Calvo, M. I., Matía, P., Rubio, M. A., & Calle-Pascual, A. L. (2014). Diabetes mellitus and abnormal glucose tolerance development after gestational diabetes: A three-year, prospective, randomized, clinical-based. *Mediterranean Lifestyle Interventional Study with Parallel Groups*. 10.1016/j.clnu.2014.09.005 10.1016/j.clnu.2014.09.005 25262459

[CR66] Philis-Tsimikas, A., Fortmann, A. L., Dharkar-Surber, S., Euyoque, J. A., Ruiz, M., Schultz, J., & Gallo, L. C. (2014). Dulce mothers: An intervention to reduce diabetes and cardiovascular risk in latinas after gestational diabetes. *Translational Behavioral Medicine,**4*(1), 18–25. 10.1007/s13142-014-0253-4 24653773 10.1007/s13142-014-0253-4 PMC3958598

[CR67] Reinhardt, J. A., Van Der Ploeg, H. P., Grzegrzulka, R., & Timperley, J. G. (2012). Implementing lifestyle change through phone-based motivational interviewing in rural-based women with previous gestational diabetes mellitus. *Health Promotion Journal of Australia,**23*(1), 5–9. 22730940 10.1071/HE12005

[CR68] Rollo, M. E., Baldwin, J. N., Hutchesson, M., Aguiar, E. J., Wynne, K., Young, A., Callister, R., Haslam, R., & Collins, C. E. (2020). The feasibility and preliminary efficacy of an ehealth lifestyle program in women with recent gestational diabetes mellitus: A pilot study. *International Journal of Environmental Research and Public Health,**17*(19), 7115. 10.3390/ijerph17197115 32998401 10.3390/ijerph17197115 PMC7579575

[CR69] Rönö, K., Stach-Lempinen, B., Klemetti, M. M., Kaaja, R. J., Pöyhönen-Alho, M., Eriksson, J. G., Koivusalo, S. B., Ahmala, C., Ahola, M., Arponen, K., Andersson, S., Eriksson, J., Haapanen, P., Himanen, K., Huvinen, E., Kaaja, R., Klemetti, M., Koivusalo, S., Kylliäinen, P., & Tiitinen, A. (2014). Prevention of gestational diabetes through lifestyle intervention: Study design and methods of a finnish randomized controlled multicenter trial (RADIEL). *BMC Pregnancy and Childbirth,**14*(1), 1–11. 10.1186/1471-2393-14-70/TABLES/5 24524674 10.1186/1471-2393-14-70/TABLES/5 PMC3928878

[CR70] Rutter, H., Savona, N., Glonti, K., Bibby, J., Cummins, S., Finegood, D. T., Greaves, F., Harper, L., Hawe, P., Moore, L., Petticrew, M., Rehfuess, E., Shiell, A., Thomas, J., White, M., RutterBChir, H. M., Savona, N., Glonti, K., Cummins, S., & Harper, L. (2017). The need for a complex systems model of evidence for public health. *The Lancet,**390*, 2602–2606. 10.1016/S0140-6736(17)31267-9 10.1016/S0140-6736(17)31267-9 28622953

[CR71] Schmidt, M. I., Duncan, B. B., Castilhos, C., Wendland, E. M., Hallal, P. C., Schaan, B. D., Drehmer, M., Costa e Forti, A., Façanha, C., Nunes, M. A., e Forti, A. C., Façanha, C., Nunes, M. A., & CostaeForti, A. (2016). Lifestyle INtervention for diabetes prevention after pregnancy (LINDA-Brasil): Study protocol for a multicenter randomized controlled trial. *BMC Pregnancy & Childbirth,**16*(1), 1–12. 10.1186/s12884-016-0851-x 27029489 10.1186/s12884-016-0851-x PMC4812654

[CR72] Schulz, K. F., Altman, D. G., & Moher, D. (2010). CONSORT 2010 statement: Updated guidelines for reporting parallel group randomised trials. *BMJ,**340*(7748), 698–702. 10.1136/BMJ.C332 10.1136/BMJ.C332 PMC2844940 20332509

[CR73] Seely, E. W., Weitzman, P. F., Cortes, D., Romero Vicente, S., & Levkoff, S. E. (2020). Development and feasibility of an app to decrease risk factors for type 2 diabetes in hispanic women with recent gestational diabetes (Hola Bebé, Adiós Diabetes): Pilot pre-post study. *JMIR Formative Research,**4*(12), e19677. 10.2196/19677 33382039 10.2196/19677 PMC7808888

[CR74] Shih, S. T. F., Davis-Lameloise, N., Janus, E. D., Wildey, C., Versace, V. L., Hagger, V., Asproloupos, D., O’Reilly, S. L., Phillips, P. A., Ackland, M., Skinner, T., Oats, J., Carter, R., Best, J. D., & Dunbar, J. A. (2014). Mothers after gestational diabetes in Australia diabetes prevention program (MAGDA-DPP) post-natal intervention: An update to the study protocol for a randomized controlled trial. *Trials,**15*(1), 259. 10.1186/1745-6215-15-259 24981503 10.1186/1745-6215-15-259 PMC4083860

[CR75] Siew, L., Mingling, C., Makama, M., & O’Reilly, S. (2021). Preventing type 2 diabetes in women with previous gestational diabetes: Reviewing the implementation gaps for health behavior change programs. *Seminars in Reproductive Medicine,**38*(3), 1–7. 10.1055/s-0040-1722315 10.1055/s-0040-1722315 33511581

[CR76] Smith, B. J., Cinnadaio, N., Cheung, N. W., Bauman, A., Tapsell, L. C., & Van Der Ploeg, H. P. (2014). Investigation of a lifestyle change strategy for high-risk women with a history of gestational diabetes. *Diabetes Research and Clinical Practice,**106*, e60–e63. 10.1016/j.diabres.2014.09.035 25451910 10.1016/j.diabres.2014.09.035

[CR77] Stith, B. J., Buls, S. M., Keim, S. A., Thung, S. F., Klebanoff, M. A., Landon, M. B., Gabbe, S. G., Gandhi, K. K., & Oza-Frank, R. (2021). Moms in motion: Weight loss intervention for postpartum mothers after gestational diabetes: A randomized controlled trial. *BMC Pregnancy and Childbirth,**21*(1), 1–10. 10.1186/S12884-021-03886-3 34187391 10.1186/S12884-021-03886-3 PMC8240610

[CR78] Stokols, D. (1996). Translating social ecological theory into guidelines for community health promotion. *American Journal of Health Promotion,**10*(4), 282–298. 10.4278/0890-1171-10.4.282 10159709 10.4278/0890-1171-10.4.282

[CR79] Sukumar, N., Dallosso, H., Saravanan, P., Yates, T., Telling, C., Shorthose, K., Northern, A., Schreder, S., Brough, C., Gray, L. J., Davies, M. J., & Khunti, K. (2018). Baby steps – a structured group education programme with accompanying mobile web application designed to promote physical activity in women with a history of gestational diabetes: Study protocol for a randomised controlled trial. *Trials,**19*(1), 682. 10.1186/s13063-018-3067-8 30541621 10.1186/s13063-018-3067-8 PMC6292087

[CR80] Tang, J. W., Foster, K. E., Pumarino, J., Ackermann, R. T., Peaceman, A. M., & Cameron, K. A. (2015). Perspectives on prevention of type 2 diabetes after gestational diabetes: A qualitative study of hispanic, African-American and white women. *Maternal and Child Health Journal,**19*(7), 1526–1534. 10.1007/s10995-014-1657-y 25421329 10.1007/s10995-014-1657-y

[CR81] Tuomilehto, J., Lindström, J., Eriksson, J. G., Valle, T. T., Hämäläinen, H., Ilanne-Parikka, P., Keinänen-Kiukaanniemi, S., Laakso, M., Louheranta, A., Rastas, M., Salminen, V., Aunola, S., Cepaitis, Z., Moltchanov, V., Hakumäki, M., Mannelin, M., Martikkala, V., Sundvall, J., & Uusitupa, M. (2001). Prevention of type 2 diabetes mellitus by changes in lifestyle among subjects with impaired glucose tolerance. *New England Journal of Medicine,**344*(18), 1343–1350. 10.1056/nejm200105033441801 11333990 10.1056/nejm200105033441801

[CR82] Vounzoulaki, E., Khunti, K., Abner, S. C., Tan, B. K., Davies, M. J., & Gillies, C. L. (2020). Progression to type 2 diabetes in women with a known history of gestational diabetes: Systematic review and meta-analysis. *The BMJ,**369*, m1361. 10.1136/bmj.m1361 32404325 10.1136/bmj.m1361 PMC7218708

[CR83] WHO. (2016). WHO guidelines on physical activity and sedentary behaviour. In *Routledge Handbook of Youth Sport*. https://www.who.int/publications/i/item/9789240015128

[CR84] Zilberman-Kravits, D., Meyerstein, N., Abu-Rabia, Y., Wiznitzer, A., & Harman-Boehm, I. (2018). The Impact of a cultural lifestyle intervention on metabolic parameters after gestational diabetes mellitus a randomized controlled trial. *Maternal and Child Health Journal,**22*(6), 803–811. 10.1007/S10995-018-2450-0 29411251 10.1007/S10995-018-2450-0

